# Wash your hands: simple measures save lives

**DOI:** 10.1186/cc10683

**Published:** 2012-03-20

**Authors:** S Macedo, GV Bispo, LA Ferreira, TO Cavalcanti, PF Rosa, C Paiva, DR De Melo, LG Rezende

**Affiliations:** 1 São Jose do Avai Hospital, Itaperuna, Brazil

## Introduction

Sepsis is a challenge for the intensive therapy unit, being the principal cause of death during hospitalization.

## Methods

We realized a longitudinal and individuated intervention authorized by the HSJA ethics committee applying the campaign 'Simple Measures Save Lives' in which 105 educational adhesives served as a guide for washing hands and flags for high-contaminated locations. A decontamination routine of monitors, control panels, fans and infusion bombs was established at each 12 hours; and continued education for the health team was intensified during the intervention. Was separated two groups, patient enrollments in periods of 45 days before and after the intervention, with more than 24 hours of hospitalization: group A with 18 patients and group B with 15 patients.

## Results

The hospital infection incidence decreased by 40% and VAP by 39.6%. Urine culture was positive in 33.3% of those patients (*n *= 5) in group A and in 16.7% (*n *= 1) in group B (a 50.1% decrease). The cultures of catheter tip were positive in 68.8% (*n *= 22) of catheters in group A, which used 32 catheters in total, and none in group B, which used 13 catheters. The sepsis incidence decreased by 39.6%. Septic shock was detected in 16.6% (*n *= 3) of patients in group A. There was a drop of the costs between groups (R4,479.28, 10.5%). The cost of campaign material was R$50.00.

## Conclusion

This intervention was a simple form to decrease the related number of infections in the neurovascular ICU, having spent irrelevant values when compared with treatment of these clinical tables.
